# Terminology binding with SNOMED CT to bridge differences in information structures: a feasibility study

**DOI:** 10.1186/s13326-026-00357-6

**Published:** 2026-07-24

**Authors:** Anna Rossander, David Wetterbro, Daniel Karlsson

**Affiliations:** 1 Karolinska Institutet, Stockholm, Sweden; 2 Region Västra Götaland, Vänersborg, Sweden; 3 Region Östergötland, Linköping, Sweden; 4 Swedish eHealth Agency, Kalmar, Sweden

**Keywords:** Informatics, Health care, Semantic interoperability, Boundary problem, SNOMED CT, Ontologies, Information model

## Abstract

**Background:**

Safe and accurate sharing of healthcare information is essential for good health and well-being. Information must be exchanged among multiple parties, making the transformation between different information structures unavoidable. It has been suggested that such a transformation could be automated if information were sufficiently structured. This study aims to explore whether terminology binding to SNOMED CT can act as a bridge between different information structures.

**Methods:**

This study employed a mixed-methods approach using two information structures from the breast cancer pathology domain. Pairs of semantically equivalent user interface terms from these structures were identified by a clinician and verified by two informaticians. The terms were considered semantically equivalent when a clinician would have used them to convey the same information. The pairs of SNOMED CT concepts bound to each of the interface terms were then compared and categorised as transformable, partially transformable, or non-transformable. Non-transformable concept pairs were analysed qualitatively, using an inductive content analysis approach, to identify underlying causes.

**Results:**

73 term pairs were identified. In 70% of these, the user interface terms were terminology bound to the same SNOMED CT concept. Among term pairs with terms bound to different concepts, 9% were fully transformable, 27% partially transformable, and 64% non-transformable. All is-a-related concept pairs were either fully or partially transformable, whereas concept pairs from different hierarchies were never transformable. Major obstacles within the same hierarchy included the use of primitive concepts and nearly identical sibling concepts. For concept pairs across different hierarchies, divergent terminology-binding strategies—such as handling of integers and “other” values—hindered transformability. Additional issues were observed with local user interface terms and SNOMED CT translations.

**Conclusions:**

Although concordance between the two structures was relatively high (70%), transformability among semantically equivalent term pairs bound to different SNOMED CT concepts was low (9%). This can be improved on the terminology-binding side through guidelines and shared terminology bindings, and on the ontology side by increasing the proportion of sufficiently defined concepts and ensuring the quality of descriptions and translations. These findings are significant for practitioners seeking to enhance interoperability, as well as for SNOMED International and researchers focusing on terminology quality and transformation approaches.

**Supplementary Information:**

The online version contains supplementary material available at 10.1186/s13326-026-00357-6.

## Background

### Difficulties sharing information

Safe and accurate sharing of healthcare information is essential for good health and well-being [1]. Increasing demands from both the public and regulators call for improved information sharing. Information needs to be exchanged between patients and healthcare providers, as well as within and across healthcare organisations, for primary use and for secondary purposes, such as research and quality improvement [2]. Facilitating information sharing across different information systems can also help organisations modernise their IT systems and address technical debt [3] without losing access to existing information [4]. To make effective use of the shared information, it must be understandable and usable by the recipient, as if it were indigenous - this is called semantic interoperability [5].

Effective information sharing that preserves meaning requires structured information [6–8]. Currently, healthcare information is structured according to a variety of standards and proprietary formats [6, 9, 10]. Although ongoing efforts aim to harmonise these structures, it is argued that unifying on a single, universal information structure will never be possible [11]. Transformation between different information structures will therefore be unavoidable. Currently, this is often done manually through mapping or re-entering information, both of which are labour-intensive and error-prone [12]. It is claimed that transformation could be automated if the information were properly structured [13]. This has been studied previously [14], but studies using real-world data are uncommon. In this work, transformation refers to the process of converting information from one structure to another while maintaining its meaning.

### Definitions

In this paper, the following terms are used: An information structure is any ordered collection of relations between terms or sets of terms; it could be a standard, a proprietary structure in a healthcare information system, or a paper form. According to ISO 13972:2022, an information model is a reusable model that expresses how different concepts relate to one another, with or without stated terms for the various parts of the model [15]. A terminology is a collection of terms for a domain [16], and an ontology is a terminology that also includes information about the included concepts’ relationships to one another [17].

### Health care information structures

Healthcare information is often complex. It includes not only simple facts such as “allergic reaction” or “appendectomy” but also additional details, such as whether it is planned, ruled out, or present in a relative. Representing all possible combinations of such information solely through terminology would lead to an unmanageable number of terms, a phenomenon known as combinatorial explosion [18]. To prevent this, healthcare information is structured using a combination of terms organised through an information model or proprietary structure. Some standard information models currently in use include openEHR, OMOP, HL7 v2 messages, and HL7 FHIR. Examples of information typically stored separately include status (e.g., planned, performed, suspected, refuted) and relationships to the record’s subject, such as “mother” in “mother has diabetes”.

### Boundary problem

However, different information structures differ in how they define the boundary between what is expressed in the information model and what is captured in the terminology. The complexities of where and how to divide information between the model and the terminology constitute the “boundary problem” [14, 19]. While differences between structures are often minor [20], they pose a significant challenge to semantic interoperability.

### SNOMED CT

SNOMED CT is a comprehensive, standardised clinical terminology system designed to enable consistent representation, sharing, and analysis of healthcare information across different settings and electronic health record systems [21]. SNOMED CT is a concept-based ontology, and each concept is described with one-to-many relationships to other concepts using description logic. These relationships allow for inferences, such as recognising that a humeral fracture is both a fracture and a disorder of the upper arm.

**Fig. 1:**
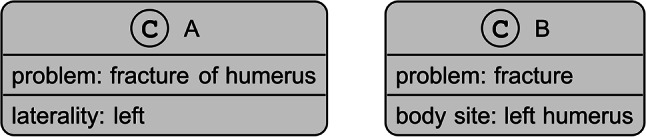
Two methods for combining structure and terminology. The same information is present in both **A** and **B**, and if they are both using SNOMED CT, it is possible to transform between the two structures

The relationships also make it possible to traverse the ontology; for example, it is possible to convert from the concepts “fracture” and “left humerus” to the concepts “fracture of the humerus” and “left” if the notion of “laterality” is also documented with SNOMED CT, see Fig. 1.

### Bridging different information structures

As discussed above, different structures define the boundary between the information model and terminology in various ways. Since an ontology can bridge differences between concepts [6], it should theoretically be possible to traverse different demarcations between the information model and terminology if both use the same ontology. This study aims to explore whether terminology binding with SNOMED CT can act as a bridge between different information structures, contributing to semantic interoperability and enhanced information sharing.

## Methods

To investigate whether terminology binding to SNOMED CT can serve as a bridge between different information structures, this study employed a mixed-methods approach with a complementary purpose [22]. First, a quantitative analysis was conducted to determine the proportion of concept pairs that were transformable; then, non-transformable concept pairs were examined qualitatively to understand why they were not.

**Fig. 2:**
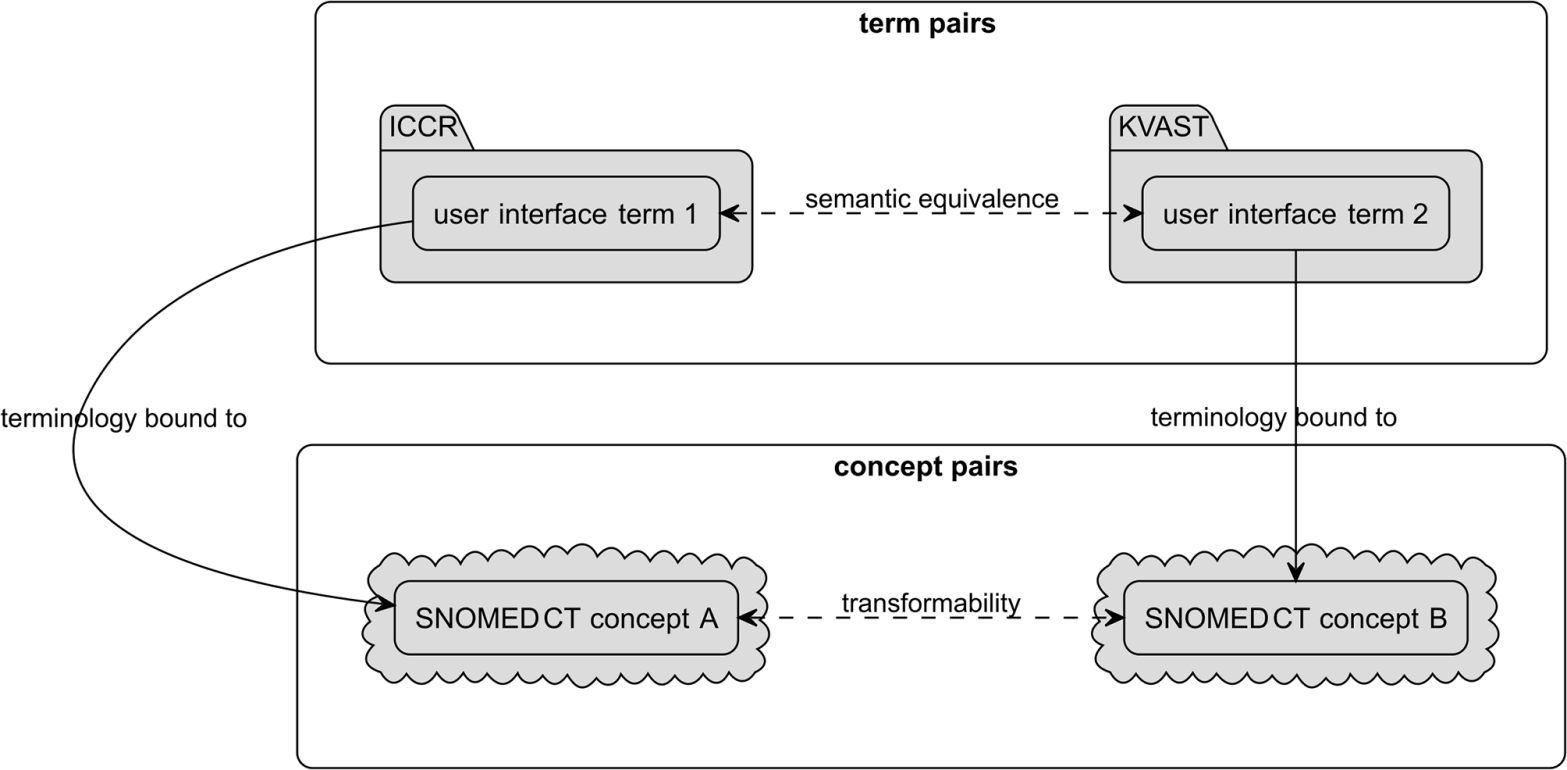
Schematic view of material showing pairs of semantically equivalent user interface terms (upper dashed arrow) and their corresponding SNOMED CT concepts (vertical arrows) that were analysed for transformability (lower dashed arrow)

Throughout this paper, we use the following terms, as illustrated in Fig. 2:

- Pairs of semantically equivalent user interface terms, further referred to as *term pairs*.
- Pairs of SNOMED CT concepts bound to each of the interface terms, further referred to as *concept pairs*.

### Material

Two information structures from the breast cancer pathology domain were used as material in this study (see Table 1). One information structure was an HL7 FHIR questionnaire version of the International Collaboration on Cancer Reporting (ICCR) breast cancer reporting form. The ICCR form had been converted into a FHIR-structured data-capture questionnaire [23], including terminology binding to SNOMED CT; similar work has been published for colorectal cancer forms [24]. For this study, the FHIR questionnaire was provided as a JSON file and used as a source.

The second information structure was developed from work carried out in a Swedish project that standardised breast cancer pathology reports [25]. In collaboration with the Swedish Society of Pathology (KVAST), the project produced a process model, a concept model, and an information model. Based on these, a Configuration Specification Spreadsheet was developed in MS Excel, with the terms within it terminology bound to SNOMED CT. The Configuration Specification Spreadsheet was used as a source for this study.

Both structures used solely pre-coordinated content; no post-coordinated expressions were used.

Neither information structure was completely finalised at the time of data collection. Since the aim of the study was to compare the transformability of structured information, not the specific information that the respective groups had structured, the available material was considered sufficient.

**Table 1:** Characteristics of the included information structures

	ICCR information structure	KVAST information structure
Input	Document template	Interviews, study visits, document templates
Initially produced structures	Terminology-bound template	Process model, Concept model and information model
Format used for this study	HL7 FHIR questionnaire	Configuration Specification Spreadsheet
Language	US English	Swedish

Some characteristics of the two included information structures

### Quantitative analysis

Concept pairs from the two information structures were categorised by type of relationship into the following categories:

- Identical.
- Non-identical.
  - related via an is-a relationship.
  - in the same hierarchy but not related via an is-a relationship.
  - from different hierarchies.

SNOMED CT International Edition 20240801 and SNOMED CT Swedish Extension 20241130 were used.

Subsequently, all but the identical concept pairs were categorised by transformability, i.e. the ability to provide machine-processable rules for transforming one representation into the other using the relationships of the concepts and the SNOMED CT concept model. Information missing in a concept compared to its counterpart was sought elsewhere within the information structure in which the concept was used. An example is given in Table 2. The assessment was performed manually, using the machine-readable concept model (MRCM) [26] as a reference.

**Table 2:** Matrix for transformability categorisation

Concept 1	Concept 2	Structure 2	Category
ABC	A	BC	Fully transformable
ABC	A	B	Partially transformable
ABC	-	-	Not transformable

Examples of the three types of transformability categories

**Fig. 3:**
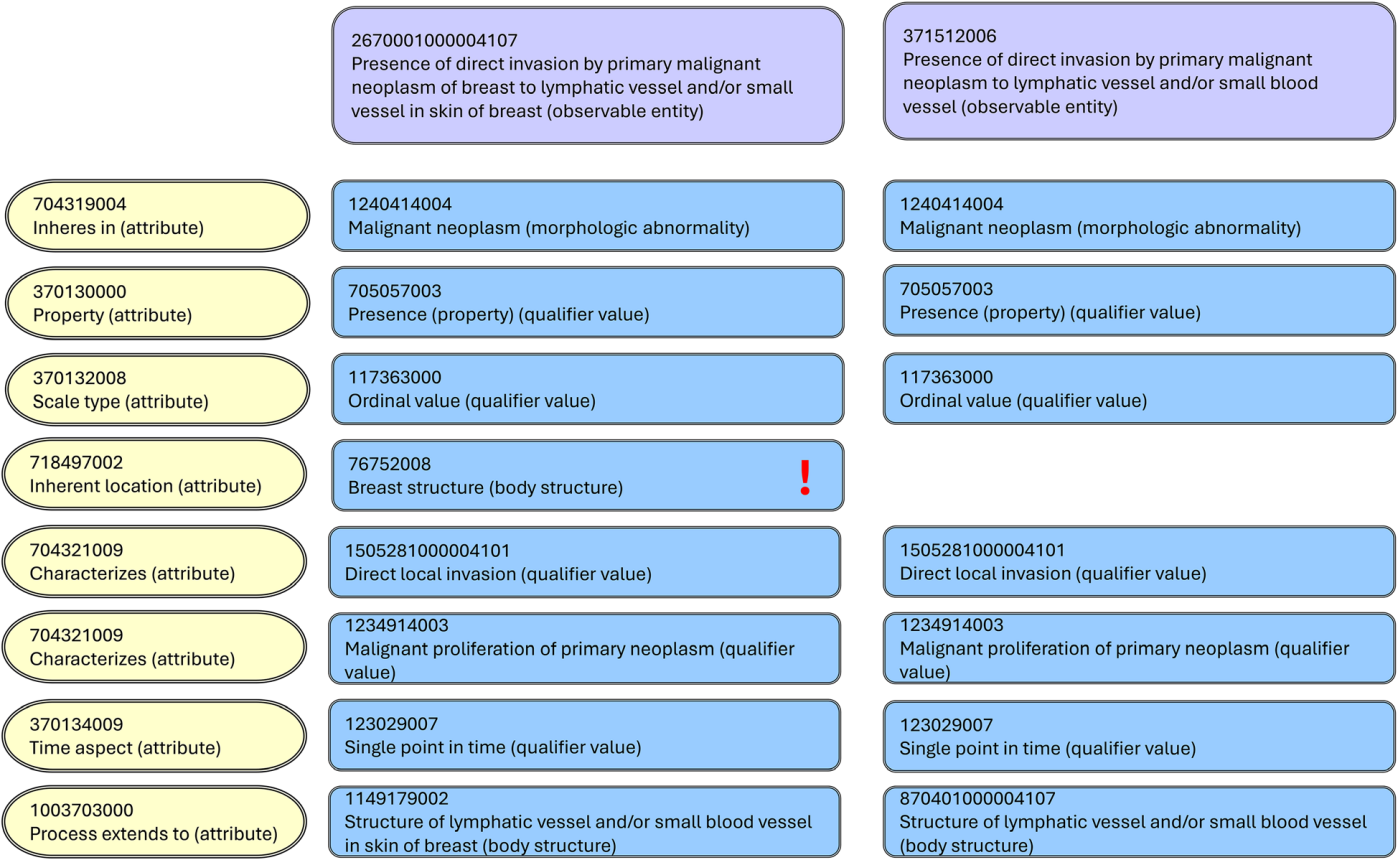
Example of a transformable concept pair. The information “breast structure” is present elsewhere within the structure

**Fig. 4:**
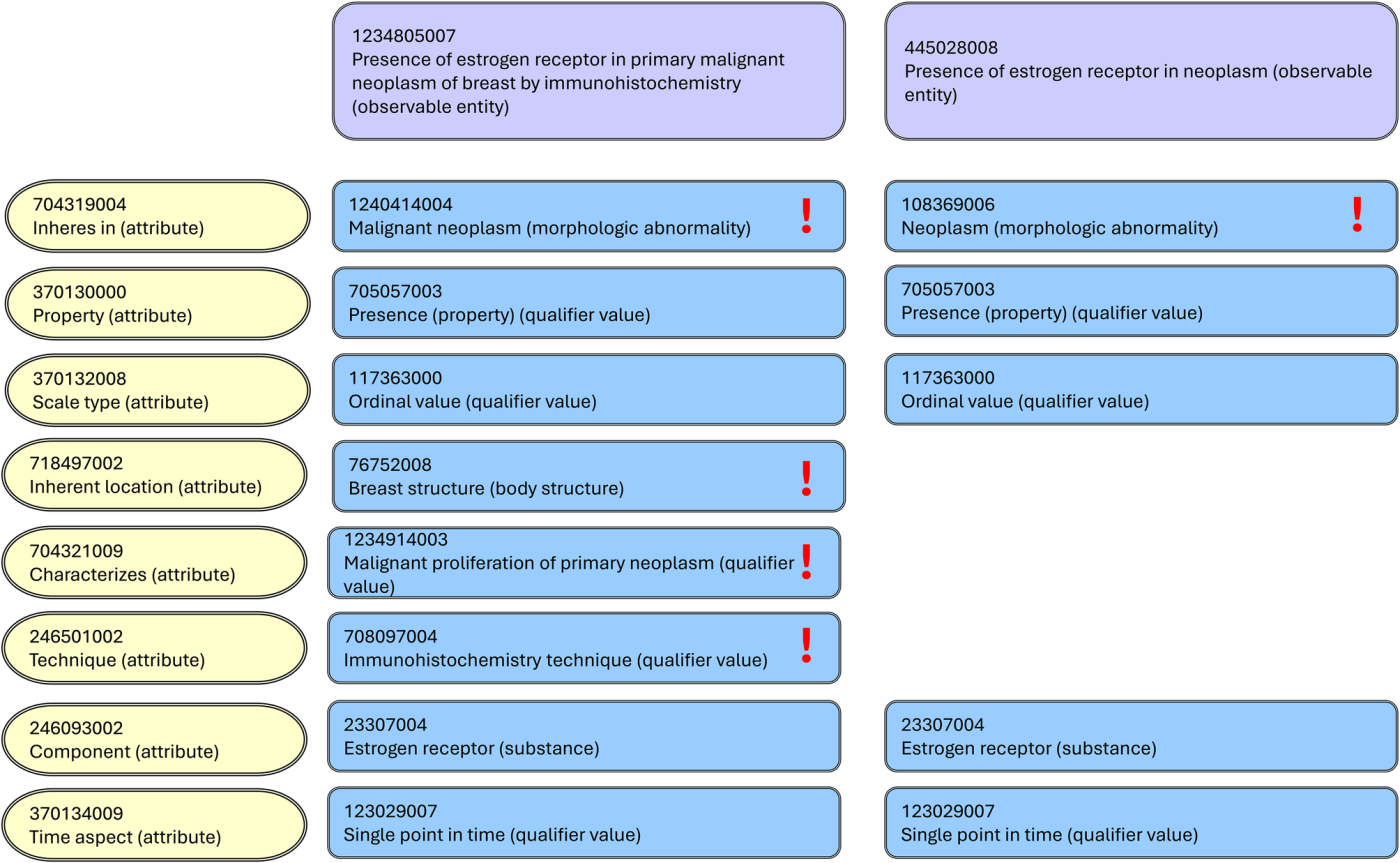
Example of a partially transformable concept pair. The information “breast structure” is present elsewhere within the structure, but “malignant” and “immunohistochemistry technique” are not

**Fig. 5:**
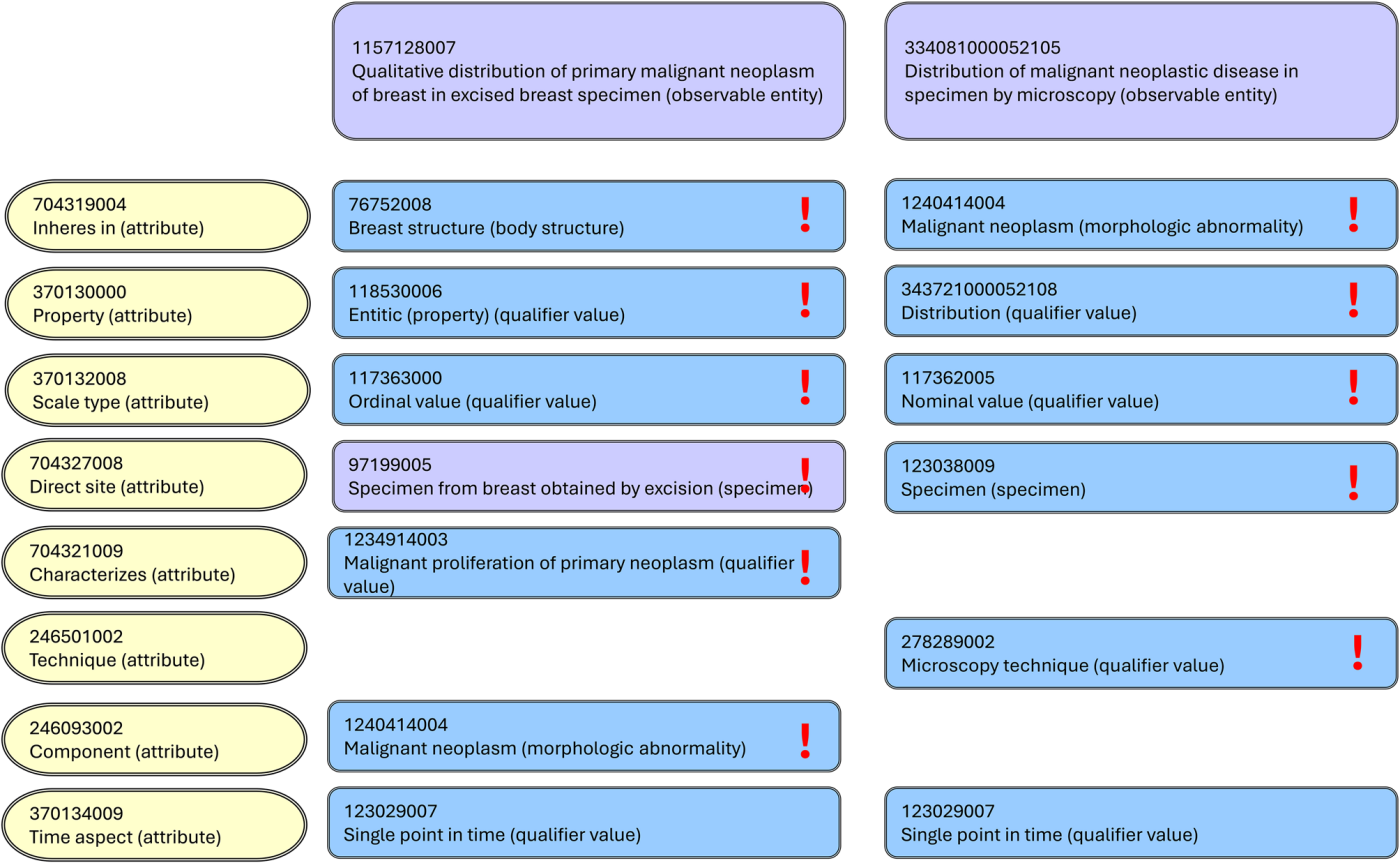
Example of a non-transformable concept pair. The two concepts are very differently modelled and not transformable via the SNOMED CT concept model, despite “breast” being present elsewhere in the structure

Concept pairs were considered transformable if the information present in the concepts and information structure together was sufficient for transformation using the SNOMED CT concept model. For example, the concepts 2670001000004107 | Presence of direct invasion by primary malignant neoplasm of breast to lymphatic vessel and/or small vessel in skin of breast (observable entity) | and 371512006 | Presence of direct invasion by primary malignant neoplasm to lymphatic vessel and/or small blood vessel (observable entity) | are regarded as transformable since the only difference is that the sample is from the breast, which is present elsewhere or can be inferred from the context of the KVAST structure, see Fig. 3. Concept pairs were considered partially transformable if additional information was found, but not sufficient for equivalence, see Fig. 4. They were regarded as non-transformable if no further information was identified, see Fig. 5.

Appendix 2 lists the 22 concept pairs and their categorisation.

### Qualitative analysis

Non-transformable concepts were analysed and grouped into categories of non-transformability, inspired by inductive content analysis [27]. All authors iteratively developed these categories while analysing the 22 non-identical concept pairs. Notes were taken in a shared datasheet, and all authors agreed upon the final categories.

### Data extraction

User interface terms for questions and answers in the ICCR FHIR questionnaire were identified in the JSON file (version 3, dated 240510). The user interface terms and SNOMED CT IDs (SCT IDs) were listed in a spreadsheet. A missing SCT ID in the source file was identified and added from a previous version. User interface terms for questions and answers in the Configuration Specification Spreadsheet by KVAST, dated 241018, were identified and added into separate columns within the same spreadsheet. The SCT ID and the Swedish preferred term (PT SE) were added.

The initial sorting was conducted by a clinician who identified similar sections of the reports based on questions, such as “estrogen receptor positivity” or “tumour size”. Both structures consisted of pathology reports and were therefore very similar in content and context. Next, an assessment was carried out of user interface terms for both questions and answers to determine whether they covered the same clinical information; if so, they were mapped to each other individually. Iteratively, semantically equivalent questions were grouped in the same row, with their answers similarly organised below, thus creating term pairs (see Fig. 2) for further analysis. Additional rows were added as necessary for answers that appeared in only one information structure.

Two informaticians evaluated the suggested term pairs, and discrepancies were resolved through discussion with both informaticians and the clinician. Questions and answers were considered semantically equivalent when it was determined that a clinician, faced with the same clinical situation in the two different information structures, would have used the matched term pairs to convey the information. Swedish and English user interface terms were used in the analyses, and all authors were proficient in both languages.

**Fig. 6:**
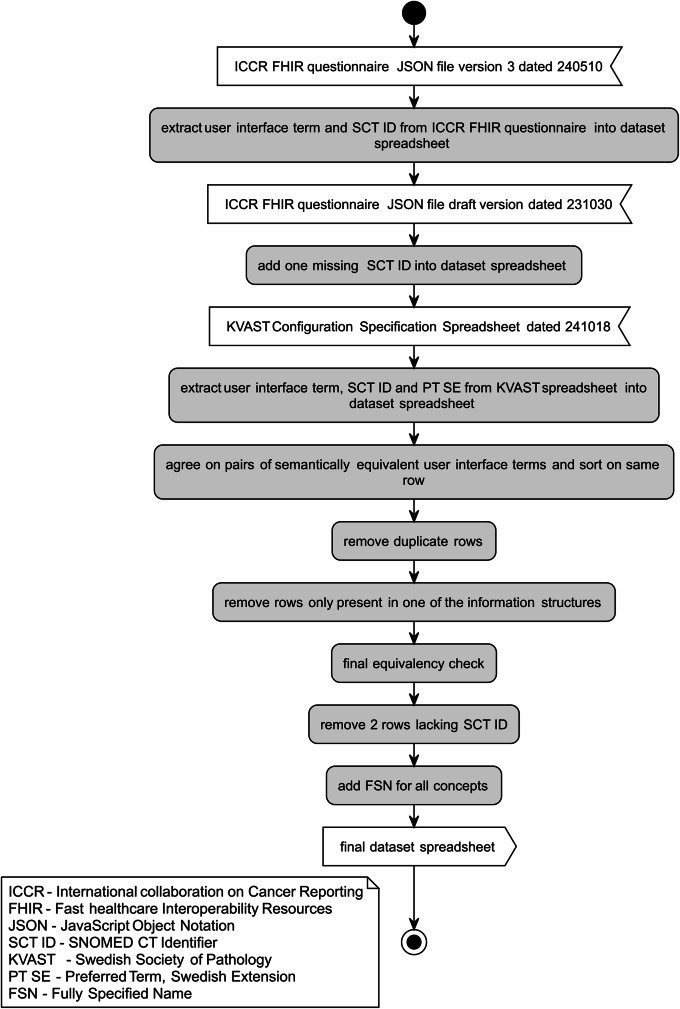
Data extraction process from the two information structures into a final dataset of pairs of semantically equivalent user interface terms and pairs of SNOMED CT concepts terminology bound to each of the interface terms

Repeated information in the material, such as the answer “not seen”, was included only once, with the remaining rows removed. Questions not addressed by either information structure, such as lymph node pathology, were omitted from the analysis. One row from each information structure was removed because no SNOMED CT concept was documented. For all remaining rows, the SNOMED CT Fully specified Name (FSN) was added, as shown in Fig. 6.

A final equivalency check was carried out by one of the informaticians, prompting renewed discussion of 9 of the suggested term pairs. The final dataset was agreed upon by consensus among the authors.

Appendix 1 shows an excerpt of the dataset spreadsheet.

### AI

AI has not been used for data collection, analysis or figure generation. ChatGPT 5 was used to assist in translating interface terms from Swedish to English for this manuscript. Grammarly (AI) was used to assist with grammar and spelling in the manuscript.

## Results

### Quantitative results

In the material, 73 pairs of semantically equivalent user interface terms were identified, 18 questions and 55 answers. There was strong consensus among authors regarding semantical equivalence. The two term pairs that lacked terminology binding to SNOMED CT in one of the information structures were assessed. One was bound to a morphological abnormality and was likely a documentation error where the binding was missing; the other was a structural header bound to an observable entity.

**Fig. 7:**
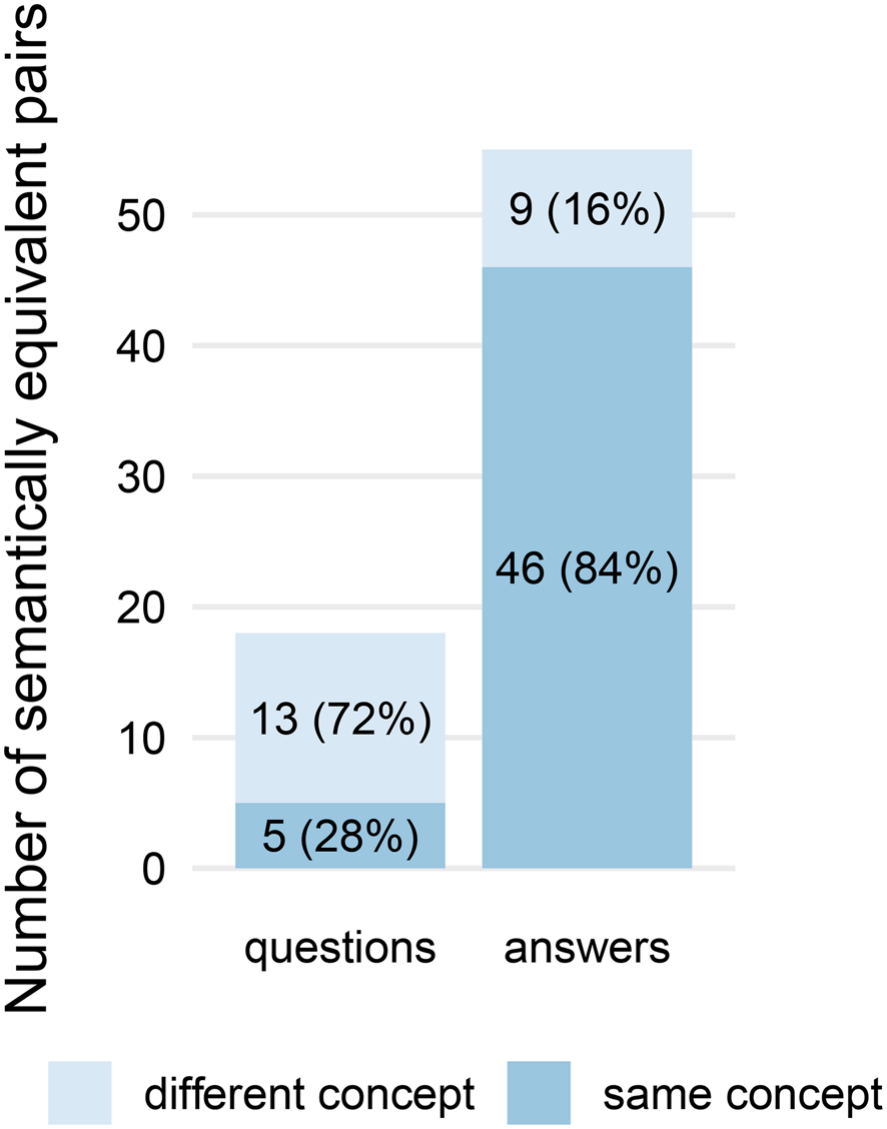
Number of semantically equivalent term pairs where the two terms were bound to the same or two different SNOMED CT concepts showed per questions and answers

In total, 70% (*n* = 51) of the term pairs were terminology bound to the same SNOMED CT concept, 28% (*n* = 5) of the questions, and 84% (*n* = 46) of the answers. Of the 22 term pairs that were terminology-bound to different SNOMED CT concepts, 13 were questions, and 9 were answers; see Fig. 7. The remaining analysis is based on these 22 term pairs and their corresponding concept pairs (see Fig. 2).

The distribution of the remaining 22 concept pairs by hierarchy, along with the definition status of the included concepts, is shown in Table 3.

**Table 3:** Distribution of hierarchies and definition status for the included concept pairs

	Total concept pairs per hierarchy	Both SD	One PRIM, one SD	Both PRIM	One PRIM and one string
Body structure	1			1	
Morphologic abnormality	2			2	
Observable entity	12	9	3	1	
Qualifier value	1			1	
Concept pairs from different hierarchies	6		1	1	3
Total concept pairs per definition status		9	4	6	3

Distribution of concept pairs across various hierarchies and the definition status of the pairs in the concepts, either primitive (PRIM) or sufficiently defined (SD)

#### Types of relationships for non-identical concept pairs

The 22 non-identical concept pairs were categorised as “related via is-a relationship”, “from the same hierarchy but not related via is-a relationship”, and “different hierarchy”; see Table 4.

**Table 4:** Proportions of relatedness in non-identical concept pairs

	Total	Is-a related	Same hierarchy, not is-a related	Different hierarchies
Questions	13	5	8	0
Answers	9	1	3	5

Types of relatedness for the 22 non-identical concept pairs

All questions were terminology-bound to concepts in the observable entity hierarchy. Answers were terminology bound to concepts from the procedure, clinical finding, morphologic abnormality, and qualifier value hierarchies. 4 answer concept pairs were from the same hierarchy, whereas 5 answer concept pairs consisted of concepts from different hierarchies.

#### Transformability

Transformability was assessed for all non-identical concept pairs. Overall, 2% of the concept pairs were transformable, 27% were partially transformable, and 64% were not transformable. See Table 5.

**Table 5:** Transformability per type of relationship between concepts in concept pairs

	Total	Is-a related	Same hierarchy not is-a related	Different hierarchies
Transformable	9% (*n* = 2)	9% (*n* = 2)	0%	0%
Partially transformable	27% (*n* = 6)	18% (*n* = 4)	9% (*n* = 2)	0%
Non-transformable	64% (*n* = 14)	0%	41% (*n* = 9)	23% (*n* = 5)

Distribution of transformability (rows) per type of relationship (columns) between concept pairs. Absolute numbers within parentheses

#### Transformable concept pairs

There were two concept pairs in which the clinical information was fully transformable, even though the concepts differed. Both pairs involved concepts connected through is-a relationships; that is, they only differed in granularity. In both cases, one concept did not include “breast” as a localisation. For example, the question regarding tumour extension localisation had an answer option “skin”, which was terminology bound to 82038008 |Skin structure of breast (body structure)| in ICCR and 39937001 |Skin structure (body structure)| in KVAST, respectively. This was entirely transformable as the contextual information “analysis for breast cancer” was present elsewhere in the KVAST information structure.

#### Partially transformable concept pairs

Partially transformable concepts that were is-a related (*n* = 4) varied in granularity regarding information not found elsewhere in the structure. Examples of such information include “primary malignant” and “immunohistochemistry”.

The two partially transformable concept pairs that were from the same hierarchy and not is-a-related (*n* = 2) lacked “primary malignant”. One pair consisted of two primitive concepts, where the existing concept should have subsumed the new one. They contained distance information and could have been sufficiently defined if a pattern for representing the distance between two landmarks had been applied. The second pair involved an older, primitive concept with no attributes and a newer, sufficiently defined concept.

#### Non-transformable concept pairs

9 concept pairs were not transformable despite belonging to the same hierarchy; 6 were from the observable entity hierarchy, 2 from the morphologic abnormality hierarchy, and 1 from the qualifier value hierarchy. The concept pairs from the observable entity hierarchy were modelled with different values for the same attribute, for example, 1157128007 |Qualitative distribution of primary malignant neoplasm of breast in excised breast specimen (observable entity)| and 334081000052105 |Distribution of malignant neoplastic disease in specimen by microscopy (observable entity)|, as also shown in Fig. 6.

2 concept pairs were from the morphologic abnormality hierarchy and shared 1187225007 |Malignant epithelial neoplasm (morphologic abnormality)| as the most proximal common parent. The final concept pair was 1156316003 |Cannot be determined (qualifier value)| and 82334004 |Indeterminate (qualifier value)|.

All 5 concept pairs that used concepts from different hierarchies were not transformable.

#### Overall semantic interoperability

**Fig. 8:**
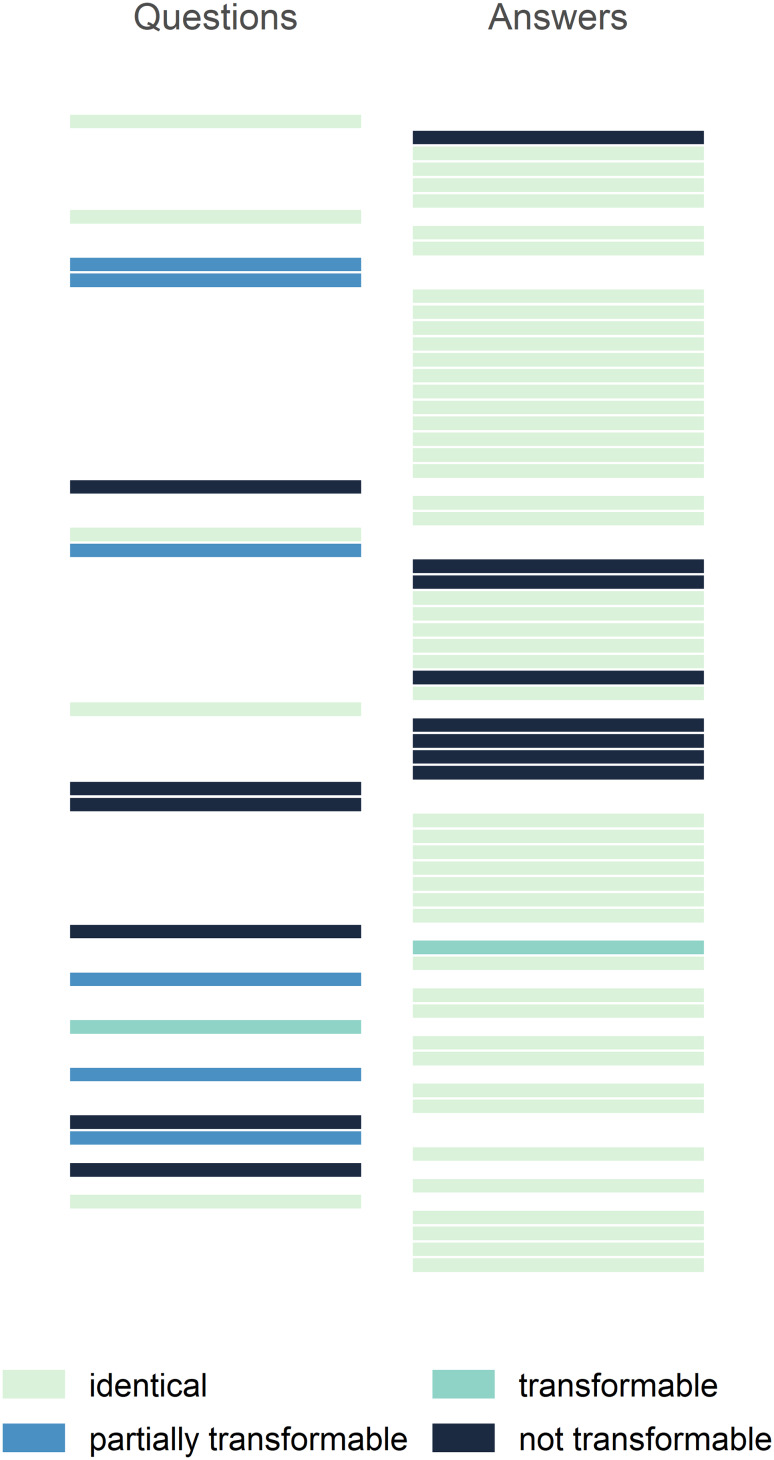
Heatmap of transformability for the 73 included concept pairs in the order of appearance in the material

Overall, terminology binding to SNOMED CT enabled semantic interoperability in 73% (*n* = 53) of cases, with 70% (*n* = 51) resulting from identical concepts and 3% (*n* = 2) employing mechanisms within the ontology to transform the information. Figure 8 illustrates the distribution of different types of transformability in the material.

### Qualitative results

Three types of non-transformability among concept pairs from different hierarchies were identified: not recorded or less-specified parent; not seen or normal and qualifier values or integers. Further, issues regarding primitive content and three types of hindrances related to concept descriptions were identified: the use of local user interface terms, incorrect translations, and near-synonyms.

#### Not recorded or less specified parent

Both information structures provided user interface terms when none of the listed answers was suitable, but employed different strategies for terminology binding these. As shown in Table 6, ICCR used the qualifier value “1220561009 |Not recorded (qualifier value)|” while KVAST used a less specific parent procedure.

**Table 6:** Example of not recorded or less specified parent

	ICCR	KVAST
Interface term question	Operative procedure performed?	Specimen from?
*Terminology bound to*	*2620001000004108 * *|Specimen collection procedure * *(observable entity)|*	*2620001000004108 * *|Specimen collection procedure * *(observable entity)|*
Interface term answer option	Not specified	Other breast surgery
*Terminology bound to*	*1220561009 * *|Not recorded (qualifier value)|*	*392090004 * *|Operation on breast (procedure)|*

Excerpt from the material showing the terminology binding of semantically equivalent information on preformed procedures in the two information structures. The material also includes additional specified procedure options that are not shown in the table for brevity

#### Not seen or normal

The two information structures employed different methods to terminology bind user interface terms for normal findings, as shown in Table 7. There is no “normal” morphologic abnormality concept in SNOMED CT. In the KVAST information structure, the user interface term was terminology bound to three concepts (as it was not yet finalised), but none was the same as the concept that was terminology bound to in the ICCR information structure. Two were from the qualifier value hierarchy, and the third was a finding. ICCR used a qualifier value.

**Table 7:** Example of not seen or normal

	ICCR	KVAST
Interface term question	Histological tumor type	Morphological type
*Terminology bound to*	*1660001000004100* *|Histologic type of primary malignant neoplasm of breast (observable entity)|*	*2100001000004103 * *|Histologic type of malignant neoplasm (observable entity)|*
Interface term answer option	No residual invasive carcinoma	Other benign finding
*Terminology bound to*	*47492008 |Not seen (qualifier value)|*	*17621005 |Normal (qualifier value)|* *or* *30807003 |Benign (qualifier value)|* *or* *30389008 |Normal tissue (finding)|*

Extract from the material showing terminology binding of semantically equivalent information on histological tumour types and normal findings in the two information structures

#### Qualifier values or integers

Both information structures terminology bound the question “histological tumour grade” to the concept 372276001 |Nottingham combined grade of primary malignant neoplasm of breast (observable entity)|. For the answers, one information structure used SNOMED CT concepts from the qualifier value hierarchy. In contrast, the other information structure used integers, see Table 8.

**Table 8:** Example of qualifier values or integers

	ICCR	KVAST
Interface term question	Histological tumor grade	NHG-grade
*Terminology bound to*	*372276001 |Nottingham combined grade of primary malignant neoplasm of breast (observable entity)|*	*372276001 |Nottingham combined grade of primary malignant neoplasm of breast (observable entity)|*
Interface term answer option	Grade 1 (scores of 3, 4, or 5)	1
*Terminology bound to*	*258351006 |Grade 1 (qualifier value)|*	-
Interface term answer option	Grade 2 (scores of 6 or 7)	2
*Terminology bound to*	*258352004 |Grade 2 (qualifier value)|*	-
Interface term answer option	Grade 3 (scores of 8 or 9)	3
*Terminology bound to*	*258353009 |Grade 3 (qualifier value)|*	-

Extract from the material showing the terminology binding of semantically equivalent information on histological tumour grades in the two information structures

#### Primitive content

Two types of primitive concepts were identified. Firstly, those where the SNOMED CT concept model is not sufficiently developed to define the concepts, such as distance observables and concepts from the qualifier value hierarchy. Secondly, there are cases where the concepts could have been defined but were not, possibly because they were included in SNOMED CT before the relevant attributes in the concept model were introduced.

#### Local user interface terms

Both information structures used local user interface terms rather than SNOMED CT descriptions. In some cases, this led to changes in meaning; for example, the user interface term “no residual invasive carcinoma” is terminology bound to the concepts 47492008 |Not seen (qualifier value)| and 385432009 |Not applicable (qualifier value)| (in different parts of the material), but it represents a stronger statement than either of them.

#### Translations

Some translations were found to be incorrect. For example, the concept 1287028000 |Histologic type of noninvasive premalignant neoplasm of breast (observable entity)| is translated as 1287028000 |histologisk grad av icke-invasiv premalign brösttumör|, which translates to English as “histological grade of non-invasive premalignant breast tumour”.

#### Near synonyms

One of the concept pairs that were non-transformable consisted of two very similar concepts: 82334004 |Indeterminate (qualifier value)| and 1156316003 |Cannot be determined (qualifier value)|. The former has a description “Not exactly known or degree or value not exactly established”, but the latter provides no additional information. Both are primitive and lack defining attributes, but share the same proximal parent, 272520006 |Degree findings (qualifier value)|.

## Discussion

### Overall semantic interoperability

The concordance of 70% (*n* = 51 of 73), i.e., the same SNOMED CT concept was chosen for both concepts in a pair of semantically equivalent user interface terms across the two data structures, can be related to intercoder reliability in studies where several coders code the same material and is higher than previous findings [28–30]. This may be due to the material’s structured nature and the well-developed pathology domain in SNOMED CT [31]. A larger dataset might yield different results and be more generalisable to SNOMED CT hierarchies that are not well represented in this sample, such as procedures, clinical findings and situations with explicit context hierarchy.

In this study, we have included each term pair only once, even though some term pairs, for example, “not present,” appear multiple times in the material. The 70% concordance thus pertains to the included types of term pairs, not the overall information structure. The focus of this study is on the transformability of the 22 non-identical concept pairs, and we have not calculated any inter-rater agreement measure, such as Cohen’s Kappa.

### Transformability in general

There was a close relationship between the distance in SNOMED CT and transformability. All concept pairs related by ‘is-a’ were fully or partially transformable, whereas concept pairs from different hierarchies were never transformable. Traversing within a hierarchy from a pre-coordinated concept to another concept refined with a post-coordinated expression is shown to be possible as long as all necessary information is present in both structures [32–34], so this finding is expected. It is also possible to transform between the clinical findings or procedures hierarchy and the Situation with explicit context hierarchy [33]. The transformation between Situation concepts and FHIR resources that contain a finding or procedure, as well as the context switch, is illustrated by a demonstrator developed by SNOMED International [35]. However, in this material, there were no concepts from the Situation with explicit context, so this was not examined here. Non-transformability was partly due to the lack of guidelines or failure to use existing ones. To our knowledge, there is no terminology binding guidance on the use of “other” or on specific data types, such as integers versus SNOMED CT qualifier values. Stricter guidelines for terminology binding could have enabled a higher degree of transformability. Additionally, modelling guidelines, for example, on distance patterns, could potentially improve transformability.

### Transformable concept pairs

Only 9% of the non-identical concept pairs were transformable via SNOMED CT. This figure is based on a sample of only 22 concept pairs, and it might differ in a different dataset, but it is nevertheless low. Transformability was only present between is-a-related concepts, i.e., differences in granularity, where one information structure had some information separately, and the other used a pre-coordinated concept.

### Partially transformable concept pairs

For partially transformable concepts that were is-a related but where information was missing, a possible solution would be to use the less detailed concept, which should be correct but less informative. The clinical effect of this has not been examined in this study. For primitive concepts, insufficient information was included in the modelling, hindering machine-based transformation, even when all necessary information was present in the FSN and the information structure.

### Non-transformable concept pairs

The non-transformable concept pairs that were from the observable entity hierarchy but not is-a related used slightly different values in their modelling, for example, “percentage” or “number fraction”, making them siblings. The observable entity model is complex to accommodate many different use cases, but this also opens the door to greater discrepancies in both terminology binding and when modelling concepts. Quality and consistency pose challenges, especially for concepts developed in extensions [36].

The remaining non-transformable concept pairs were from hierarchies with a less complex concept model: morphologic abnormality and qualifier values. The lack of attributes makes them inherently non-transformable using SNOMED CT logic. A solution to this is to meet at the most proximal common parent, which might be sufficient depending on the ontology’s depth. At the time of writing, a quality initiative is ongoing in the morphological abnormality hierarchy, which might add more is-a relationships, making it a feasible solution for those concept pairs [37].

#### Not recorded or less specified parent

There is a discrepancy between “not elsewhere classified” and “not specified”: the former is related to the other entries in the structure, whereas the latter simply says that there is no more information [38]. The term “other” is frequently used in health care information systems as a final option in a list of options, without clearly stating if it means “any type, excluding the others in the list” (not elsewhere classified) or “any type, including but not limited to the others in the list” (not specified).

One pair of semantically equivalent user interface terms was “other breast surgery” and “not specified”. Since the word “other” is open to interpretation, they potentially could represent two different things and then not be semantically equivalent. However, for the users of the two cancer reports, we say that these two options would be chosen in the same clinical situation and thus should be equivalent. The ICCR material was terminology-bound to 1220561009 |Not recorded (qualifier value)|, which is a NULL value. In contrast, the KVAST material was terminology-bound to a proximal parent, 392090004 |Operation on breast (procedure)|, probably because there is a recommendation in Sweden to use a proximal parent for terminology binding “not specified” [39]. To our knowledge, there is no internationally agreed-upon advice on this. An advantage of using a less specified concept is that it enables reasoning over the recorded data even without knowledge of the other options in the user interface, and it supports semantic interoperability between structures with different value sets.

#### Not seen or normal

The difference in terminology between “no residual invasive carcinoma” and “other benign finding” is partly due to medicolegal discrepancies between Sweden and the USA. The ICCR term was bound to 47492008 |Not seen (qualifier value)|, indicating that the sought-for pathology may be present in the specimen but has not been identified. In contrast, all three considered options in the KVAST material stated that the specimen was normal.

#### Qualifier value or integers

Both qualifier values representing a number, as “258351006 |Grade 1 (qualifier value)|” and integers as “1”, are possible to use according to the OWL2 flavour that SNOMED CT uses [40], but are not transformable between each other via the MRCM. A machine-processable rule could likely be developed to handle different settings and value types.

#### Primitive content

Some of the partially transformable concept pairs would have been transformable had they been sufficiently defined. Overall, 39% of the concepts in the International Edition were defined (40% as of 20260101), but in the observable entity hierarchy, this figure is just 15%. Quality assurance work is ongoing within SNOMED International, and research has examined ways to facilitate this with machine support, primarily focusing on identifying missing concepts [41].

#### Local user interface terms and translations

Local interface terms introduce an additional step between the users of the information and may lead to semantic drift in the concept’s meaning. Forcefully using only SNOMED CT descriptions (terms) might, however, result in an unruly user interface, and this is probably why both the information structures in this study and similar work within colorectal cancer [31] use local user interface terms. Also, as our results show, even translations provided within SNOMED CT might be erroneous and introduce semantic drift. Quality-assuring descriptions for SNOMED CT concepts is a possible area for future work to make SNOMED CT safer and more usable [4].

#### Near synonyms

The non-transformable qualifier value concept pair consists of two very similar entities, “Cannot be determined” and “Indeterminate”, where one could argue that the first alludes more to the process and the second to the examination’s output. This might be more intuitive for native English speakers than other users of SNOMED CT. Perhaps this could be clarified by adding a description of the type definition [42] for the concepts to support more stringent terminology binding. Another option would be to inactivate one concept and refer to the other. Sharing terminology bindings publicly could also help different groups select the same SNOMED CT concept for a given clinical idea.

## Conclusions

In this study, we have investigated whether SNOMED CT delivers on its promise of enabling the transformation of information across different information structures. We found a 70% concordance between the two structures, which is high. However, transformability among pairs of semantically equivalent user interface terms bound to different SNOMED CT concepts was low (9%).

This can be improved on the terminology-binding side by guidelines and shared terminology bindings, and on the ontology side by increasing the proportion of defined concepts and by quality assurance of descriptions and translations. These findings support practitioners in expanding the use of guidelines and sharing work, and guide SNOMED International and researchers in focusing on quality improvement areas and transformation methods.

## Electronic Supplementary Material

Below is the link to the electronic supplementary material.

Supplementary Material 1

Supplementary Material 2

## Data Availability

The datasets supporting the conclusions of this article are available in the Zenodo repository, (10.5281/zenodo.18196967).

## Abbreviations

CIMI: Clinical Information Modelling Initiative
EHDS: European Health Data Space
FHIR: Fast Healthcare Interoperability Resources
FSN: Fully Specified Name
HL7: Health Level Seven, an international standards developing organisation within healthcare
ICCR: International Collaboration on Cancer Reporting
JSON: JavaScript Object Notation
KVAST: Swedish Society of Pathology
MRCM: machine-readable concept model (for SNOMED CT)
OHDSI: Observational Health Data Sciences and Informatics
OMOP: Observational Medical Outcomes Partnership (OMOP) Common Data Model (CDM)
openEHR: a non‑profit organisation that publishes technical standards for an EHR platform, along with domain-developed clinical models to define content
PT SE: Preferred Term, Swedish Extension
SCT ID: SNOMED CT Identifier
SNOMED CT: SNOMED CT clinical terminology

## References

[1] American Hospital Association. Sharing data, saving lives: the hospital agenda for interoperability. 2019. https://www.aha.org/system/files/2019-01/Report01_18_19-Sharing-Data-Saving-Lives_FINAL.pdf. Accessed 27 Oct 2025.

[2] Goldacre B, Morley J, Hamilton N, Better. Broader, safer: using health data for research and analysis. 2022. https://www.gov.uk/government/publications/better-broader-safer-using-health-data-for-research-and-analysis. 10.1088/1361-6498/ac89f8 35973413

[3] Kruchten P, Nord RL, Ozkaya I. Technical debt: from metaphor to theory and practice. IEEE Softw. 2012;29:18–21. https://doi.org/10/gd55m2.

[4] Rossander A, Lindsköld L, Ranerup A, Karlsson D. A State-of-the Art Review of SNOMED CT Terminology Binding and Recommendations for Practice and Research. Methods Inf Med. 2021;60:e76–88. 10.1055/s-0041-1735167. 34583415 10.1055/s-0041-1735167 PMC8714300

[5] European Commission. European interoperability framework - implementation strategy annex 2. https://eur-lex.europa.eu/legal-content/EN/TXT/HTML/?uri=CELEX:52017DC0134

[6] De Mello BH, Rigo SJ, Da Costa CA, Da Rosa Righi R, Donida B, Bez MR, et al. Semantic interoperability in health records standards: a systematic literature review. Health Technol. 2022;12:255–72. 10.1007/s12553-022-00639-w. 10.1007/s12553-022-00639-w PMC8791650 35103230

[7] Wilkinson MD, Dumontier M, Aalbersberg IJ, Appleton G, Axton M, Baak A, et al. The FAIR Guiding Principles for scientific data management and stewardship. Sci Data. 2016;3:160018. 10.1038/sdata.2016.18. 26978244 10.1038/sdata.2016.18 PMC4792175

[8] Lin AY, Arabandi S, Beale T, Duncan WD, Hicks A, Hogan WR, et al. Improving the Quality and Utility of Electronic Health Record Data through Ontologies. Standards. 2023;3:316–40. 10.3390/standards3030023. 37873508 10.3390/standards3030023 PMC10591519

[9] Schulz S, Stegwee R, Chronaki C. Standards in Healthcare Data. In: Kubben P, Dumontier M, Dekker A, editors. Fundamentals of Clinical Data Science. Cham: Springer International Publishing; 2019. pp. 19–36. 10.1007/978-3-319-99713-1_3. 31314244

[10] Tsafnat G, Dunscombe R, Gabriel D, Grieve G, Reich C. Converge or Collide? Making Sense of a Plethora of Open Data Standards in Health Care. J Med Internet Res. 2024;26:e55779. 10.2196/55779. 38593431 10.2196/55779 PMC11040436

[11] Martínez-Costa C, Karlsson D, Schulz S. Ontology patterns for clinical information modelling. In: Proceedings of the 5th International Conference on Ontology and Semantic Web Patterns - Volume 1302. Riva del Garda, Italy: CEUR-WS.org; 2014. p. 61–72. 10.5555/2878937.2878944.

[12] Michel J, Hsiao A, Fenick A. Using a scripted data entry process to transfer legacy immunization data while transitioning between electronic medical record systems. Appl Clin Inf. 2014;5:284–98. 10.4338/ACI-2013-11-RA-0096. 10.4338/ACI-2013-11-RA-0096 PMC3974261 24734139

[13] Kalwar SSMART. Towards automated mapping between data specifications. In: 33rd International Conference on Software Engineering & Knowledge Engineering (SEKE 2021): Proceedings. 2021. p. 429–36. 10.18293/SEKE2021-161.

[14] Markwell D, Sato L, Cheetham E. Representing clinical information using SNOMED clinical terms with different structural information models. In: Proceedings of the Third International Conference on Knowledge Representation in Medicine. Phoenix, Arizona, USA; 2008. https://ceur-ws.org/Vol-410/Paper13.pdf

[15] International Organization for Standardization. Health informatics — Clinical information models — Characteristics, structures and requirements (ISO 13972:2022). Geneva; 2022.

[16] International Organization for Standardization. Terminology work and terminology science — Vocabulary (ISO 1087:2019). Geneva; 2019.

[17] Ghomari LZ, Ghomari AR. Ontology versus terminology, from the perspective of ontologists. Int J Web Sci. 2012;1:315. 10.1504/IJWS.2012.052531.

[18] Rector AL. Clinical Terminology: Why Is it so Hard? Methods Inf Med. 1999;38:239–52. 10.1055/s-0038-1634418. 10805008

[19] Martínez-Costa C, Schulz S. Validating EHR clinical models using ontology patterns. J Biomed Inf. 2017;76:124–37. 10.1016/j.jbi.2017.11.001. 10.1016/j.jbi.2017.11.001 29113934

[20] Rossander A, Karlsson D. Structure of Health Information With Different Information Models: Evaluation Study With Competency Questions. JMIR Med Inf. 2023;11:e46477. 10.2196/46477. 10.2196/46477 PMC10425817 37523221

[21] SNOMED International. https://www.snomed.org/. Accessed 13 Jan 2026.

[22] Venkatesh V, Brown SA, Bala H. Bridging the Qualitative-Quantitative Divide: Guidelines for Conducting Mixed Methods Research in Information Systems. MIS Q. 2013;37:21–54. 10.25300/MISQ/2013/37.1.02.

[23] HL7 FHIR Questionnaire Resource. https://www.hl7.org/fhir/questionnaire.html

[24] Hwang J, Goel AK, Rous BA, Birdsong G, Seegers PA, Dubois S, et al. Building a Standardized Cancer Synoptic Report With Semantic and Syntactic Interoperability: Development Study Using SNOMED CT and Fast Healthcare Interoperability Resources (FHIR). JMIR Med Inf. 2025;13:e76870–76870. 10.2196/76870. 10.2196/76870 PMC12463337 40997319

[25] Insatsområde strukturerad vårdinformation patologi. https://skr.se/kunskapsstyrningvard/programomradenochsamverkansgrupper/nationellasamverkansgrupper/nsgstruktureradvardinformation/struktureradvardinformationpatologi.56159.html. Accessed 24 Apr 2024.

[26] SNOMED International. SNOMED CT machine readable concept model. https://confluence.ihtsdotools.org/display/DOCMRCM/SNOMED+CT+Machine+Readable+Concept+Model.

[27] Lyhne CN, Thisted J, Bjerrum M. Qualitative content analysis–framing the analytical process of inductive content analysis to develop a sound study design. Qual Quant. 2025;59:5329–49. 10.1007/s11135-025-02220-9.

[28] Chiang MF, Hwang JC, Yu AC, Casper DS, Cimino JJ, Starren JB. Reliability of SNOMED-CT coding by three physicians using two terminology browsers. In: AMIA Annual Symposium proceedings. 2006. p. 131–5. https://pmc.ncbi.nlm.nih.gov/articles/PMC1839418/pdf/AMIA2006_0131.pdf PMC1839418 17238317

[29] Schulz S, Daumke P, Romacker M, López-García P. Representing oncology in datasets: Standard or custom biomedical terminology? Inf Med Unlocked. 2019;15:100186. 10.1016/j.imu.2019.100186.

[30] Vorisek CN, Klopfenstein SAI, Sass J, Lehne M, Schmidt CO, Thun S. Evaluating suitability of SNOMED CT in structured searches for COVID-19 studies. Stud Health Technol Inform. IOS; 2021. 10.3233/SHTI210126. 10.3233/SHTI210126 34042711

[31] Campbell WS, Rous BA, Dubois S, Seegers PA, Dash RC, Rüdiger T, et al. Advancements in interoperability: achieving anatomic pathology reports that adhere to international standards and are both human-readable and readily computable. JCO Clin Cancer Inf. 2025;e2400180. 10.1200/CCI-24-00180. 10.1200/CCI-24-00180 39908464

[32] Arguello-Casteleiro M, Martínez-Costa C, Des-Diz J, Maroto N, Fernandez-Prieto MJ, Stevens R, From SNOMED CT expressions to an FHIR RDF representation: exploring the benefits of an ontology-based approach. In: Proceedings of the Joint Ontology Workshops (CEUR-WS Vol. 2518). 2019. p. 13. https://ceur-ws.org/Vol-2518/paper-ODLS1.pdf

[33] Martinez-Costa C, Cornet R, Karlsson D, Schulz S, Kalra D. Semantic enrichment of clinical models towards semantic interoperability. The heart failure summary use case. J Am Med Inf Assoc. 2015;565–76. 10.1093/jamia/ocu013. 10.1093/jamia/ocu013 PMC11737585 25670758

[34] Practical Guide to Postcoordination | SNOMED international documents. https://docs.snomed.org/snomed-ct-practical-guides/snomed-ct-postcoordination-guide. Accessed 6 Jan 2026.

[35] Context Demonstrator | SNOMED CT implementation demos. https://ihtsdo.github.io/sct-implementation-demonstrator/context. Accessed 6 Jan 2026.

[36] Lee DH, Lau FY. An exploratory analysis of SNOMED CT national editions. J Am Med Inf Assoc. 2025;ocaf184. 10.1093/jamia/ocaf184. 10.1093/jamia/ocaf184 PMC12844573 41133785

[37] Histology Content Quality Project Group - SNOMED Spaces. https://conf.spaces.snomed.org/wiki/spaces/HCQG/overview. Accessed 7 Jan 2026.

[38] Cimino JJ. Desiderata for Controlled Medical Vocabularies in the Twenty-First Century. Methods Inf Med. 1998;37:394–403. https://doi.org/10/ggwp2q PMC3415631 9865037

[39] NAG användning av Snomed CT. Ospecificerat och annan vid urval ur Snomed CT. 2022. https://kunskapsstyrningvard.se/download/18.2b5d80d81891f93eaff64e1f/1689154605208/Ospecificerat-och-annan-vid-urval-Snomed-CT-v1.0.pdf. Accessed 7 Nov 2025.

[40] SNOMED CT Logical Profile Specification | SNOMED international documents. https://docs.snomed.org/snomed-ct-specifications/snomed-ct-logical-profile-specification/introduction/1-introduction. Accessed 8 Jan 2026.

[41] Hao X, Abeysinghe R, Roberts K, Cui L. Logical definition-based identification of potential missing concepts in SNOMED CT. BMC Med Inf Decis Mak. 2023;23:87. 10.1186/s12911-023-02183-7. 10.1186/s12911-023-02183-7 PMC10169302 37161566

[42] textual definition | SNOMED CT Glossary | SNOMED international documents. https://docs.snomed.org/snomed-international-documents/snomed-ct-glossary/t/textual-definition. Accessed 8 Jan 2026.

