# Supplementary material for: Terminology binding with SNOMED CT to bridge differences in information structures: a feasibility study
Source: J Biomed Semantics. 2026 Jul 24;17:13. doi: 10.1186/s13326-026-00357-6 (PMC13401298; doi:10.1186/s13326-026-00357-6)
Supplement: Supplementary file 1 — Supplementary Material 1 [file 13326_2026_357_MOESM1_ESM.pdf]

ICCR user interface terms			Terminology bound to		KVAST user interface terms		Terminology bound to			Included in final data set
Question	Answer level 1	Answer level 2	SCT ID	SCT FSN	Question	Answer	SCT ID	SCT PT SE	SCT FSN	
Operative procedure performed?			2620001000004108	Specimen collection procedure (observable entity)	Preparat från		2620001000004108	provtagning	Specimen collection procedure (observable entity)	yes
	Not specified		1220561009	Not recorded (qualifier value)		Annan bröstkirurgi	392090004	operation av bröst	Operation on breast (procedure)	yes
	Excision (less than total mastectomy)		1231734007	Excision of breast (procedure)						no
		Diagnostic excision/ Excision biopsy/localisation biopsy	1231734007	Excision of breast (procedure)		Lokal excision	1231734007	excision av bröst	Excision of breast (procedure)	yes
		Therapeutic wide local excision	237371007	Wide local excision of breast lesion (procedure)		Utvidgad excision	237371007	lokal excision med vida marginaler av bröstlesion	Wide local excision of breast lesion (procedure)	yes
		Duct excision/ Microdochectomy	237400005	Microdochectomy (procedure)						no
		Re-excision	395165008	Re-excision of breast for clearance of tumor margins (procedure)						no
						Sektorresektion	41104003	kilexcision från bröst	Wedge excision of breast (procedure)	no
