# Supplementary material for: Terminology binding with SNOMED CT to bridge differences in information structures: a feasibility study
Source: J Biomed Semantics. 2026 Jul 24;17:13. doi: 10.1186/s13326-026-00357-6 (PMC13401298; doi:10.1186/s13326-026-00357-6)
Supplement: Supplementary file 2 — Supplementary Material 2 [file 13326_2026_357_MOESM2_ESM.pdf]

ICCR user interface terms		Terminology bound to		KVAST user interface terms		Terminology bound to				
Question	Answer	SCT ID	SCT FSN	Question	Answer	SCT ID	SCT PT SE	SCT FSN	Categorisation	Transformability
	Not specified	1220561009	Not recorded (qualifier value)		Annan bröstkirurgi	392090004	operation av bröst	Operation on breast (procedure)	different hierarchy	non-transformable
Distance from nipple		1240401000	Distance of primary malignant neoplasm of breast to nipple in excised breast specimen (observable entity)	Avstånd från mamill		1290349008	avstånd mellan brösttumör och bröstvårta i exciderat prov från bröst	Distance of neoplasm of breast to nipple in excised breast specimen (observable entity)	not is-a	partially transformable
Clock position		3980001000004105	Radial location of primary malignant neoplasm in excised breast specimen (observable entity)	Klockslag		443772002	tumörposition på tänkt urtavla	Clockface position of neoplasm (observable entity)	not is-a	partially transformable
Tumor focality		1157128007	Qualitative distribution of primary malignant neoplasm of breast in excised breast specimen (observable entity)	Fokalitet		334081000052105	Distribution of malignant neoplastic disease in specimen by microscopy (observable entity)	Distribution of malignant neoplastic disease in specimen by microscopy (observable entity)	not is-a	non-transformable
Histological tumor type		1660001000004100	Histologic type of primary malignant neoplasm of breast (observable entity)	Morfologisk typ		2100001000004103	malign tumör, histologisk typ	Histologic type of malignant neoplasm (observable entity)	is-a	partially transformable
	No residual invasive carcinoma	47492008	Not seen (qualifier value)		Annat benignt fynd	17621005, 30807003, 30389008	normal, benign, normal vävnad	Normal (qualifier value), Benign (qualifier value), Normal tissue (finding)	different hierarchy	non-transformable
	Invasive breast carcinoma of no special type (invasive ductal carcinoma, not otherwise specified)	82711006	Infiltrating duct carcinoma (morphologic abnormality)		Invasiv bröstcancer (UNS)	1187332001	adenokarcinom	Adenocarcinoma (morphologic abnormality)	not is-a	non-transformable
	Carcinoma with apocrine differentiation	1187425009	Carcinoma (morphologic abnormality)		Invasiv cancer med apokrin differentiering	57141000	Apocrine adenocarcinoma (morphologic abnormality)	Apocrine adenocarcinoma (morphologic abnormality)	not is-a	non-transformable
	Grade 1 (scores of 3, 4, or 5)	258351006	Grade 1 (qualifier value)		1	-			different hierarchy	non-transformable.
	Grade 2 (scores of 6 or 7)	258352004	Grade 2 (qualifier value)		2	-			different hierarchy	non-transformable.
	Grade 3 (scores of 8 or 9)	258353009	Grade 3 (qualifier value)		3	-			different hierarchy	non-transformable.
	Score cannot be determined	1156316003	Cannot be determined (qualifier value)		grad ej bedömbar	82334004	obestämbar	Indeterminate	not is-a	non-transformable
Carcinoma in situ		1240396004	Presence of ductal carcinoma in situ in excised breast specimen (observable entity)	Finns cancer in situ i anslutning till den invasiva tumören?		1095781000004107	förekomst av carcinoma in situ vid resektionskant i exciderat vävnadsprov	Presence of carcinoma in situ at surgical margin in excised tissue specimen (observable entity)	not is-a	non-transformable
Type of carcinoma in situ		1287028000	Histologic type of noninvasive premalignant neoplasm of breast (observable entity)	Morfologisk typ		2100001000004103	malign tumör, histologisk typ	Histologic type of malignant neoplasm (observable entity)	not is-a	non-transformable
Tumor extension		3280001000004106	Anatomic location directly invaded by primary malignant neoplasm of breast (observable entity)	Invasion utanför bröstparenkymet		396464009	förekomst av tumör vid parenkymal resektionskant i exciderat vävnadsprov	Presence of neoplasm at surgical parenchymal margin in excised tissue specimen (observable entity)	not is-a	non-transformable.
	Satellite skin foci of invasive carcinoma are present (i.e., not contiguous with the invasive carcinoma in the breast) (classified as pT4b)	82038008	Skin structure of breast (body structure)		Invasion i hud	39937001	hud	Skin structure (body structure)	is-a	transformable
Invasive carcinoma		3060001000004102	Presence of primary carcinoma at surgical margin in excised specimen of breast (observable entity)	Tumören radikalt borttagen?		371488000	förekomst av tumör vid exciderad resektionskant	Presence of neoplasm at excised surgical margin (observable entity)	is-a	partially transformable
Lymphovascular invasion in primary breast carcinoma		2670001000004107	Presence of direct invasion by primary malignant neoplasm of breast to lymphatic vessel and/or small vessel in skin of breast (observable entity)	Kärlinvasion		371512006	förekomst av direkt inväxt av primär malign tumör till lymfkärl och/eller litet blodkärl	Presence of direct invasion by primary malignant neoplasm to lymphatic vessel and/or small blood vessel (observable entity)	is-a	transformable
Estrogen receptor results		1234805007	Presence of estrogen receptor in primary malignant neoplasm of breast by immunohistochemistry (observable entity)	Bedömning av ER-status		445028008	förekomst av östrogenreceptor i tumör	Presence of estrogen receptor in neoplasm (observable entity)	is-a	partially transformable
Percent of cells with nuclear positivity		1234804006	Percent of cells with estrogen receptor in primary malignant neoplasm of breast by immunohistochemistry (observable entity)	Östrogenreceptor (ER)		444644009	andel östrogenreceptorer i tumör med immunfärgning	Number fraction of estrogen receptors in neoplasm using immune stain (observable entity)	not is-a	non-transformable.
Progesterone receptor results		1234801003	Presence of progesterone receptor in primary malignant neoplasm of breast by immunohistochemistry (observable entity)	Bedömning av PR-status		445029000	förekomst av progesteronreceptor i tumör	Presence of progesterone receptor in neoplasm (observable entity)	is-a	partially transformable
Percent of cells with nuclear positicity		1234803000	Percent of cells with progesterone receptor in primary malignant neoplasm of breast by immunohistochemistry (observable entity)	Progesteronreceptor (PR)		445366002	andel progesteronreceptorer i tumör med immunfärgning	Number fraction of progesterone receptors in neoplasm using immune stain (observable entity)	not is-a	non-transformable
